# The Burden of Childhood Iron Deficiency Anemia in a Developed Country: A Croatian Tertiary Care Center Experience

**DOI:** 10.7759/cureus.50428

**Published:** 2023-12-13

**Authors:** Nusa Matijasic Stjepovic, Izabela Kranjcec, Domagoj Buljan, Helena Živić, Petra Bukovec, Martina Slukan

**Affiliations:** 1 Department of Oncology and Hematology, Children's Hospital Zagreb, Zagreb, HRV; 2 Department of Pediatrics, Children's Hospital Zagreb, Zagreb, HRV; 3 Department of General and Preventive Pediatrics, Clinic for Pediatric Medicine Helena, Zagreb, HRV; 4 Department of Pediatrics, General Hospital Varaždin, Varaždin, HRV

**Keywords:** tertiary care, primary health care, iron deficiency, child, anemia

## Abstract

Background: Given the high prevalence of unrecognized iron deficiency anemia (IDA) in Croatia and its negative impact on children’s somatic and neurological outcomes, a comprehensive preventive and treatment approach is a necessity.

Methods: This was an observational, cross-sectional study of pediatric patients referred to the Children's Hospital Zagreb, Croatia, from 2017 to 2021, for IDA. Epidemiological and clinical data were extracted. Laboratory workup and therapeutic actions in the primary and tertiary care settings were recorded. The need for transfusion, parenteral iron therapy, and hospital admission was noted.

Results: A total of 299 patients (52.2% female, median five years) were seen by the hematologist in the five-year study period. Almost half (45.1%) were referred by the primary care pediatrician. Only half of the patients (56.6%) received oral iron therapy prior to referral. The preferred preparation was Dextriferron (67.7%) during the mean period of 5.8 months, but more than one-third of the patients (36.5%) were non-compliant. Every 10th child seen by the hematologist for IDA was admitted to the hospital; 6.4% required transfusion therapy, and in only one patient, parenteral iron was administered.

Conclusions: The results of this survey established that IDA still represents an excessive burden in a tertiary care setting of a high-income country. Therefore, consistent implementation of national guidelines and additional education of primary healthcare providers is crucial to ameliorate this significant public health concern.

## Introduction

The global prevalence of anemia in 2010 was 32.9%, with iron deficiency being the most common underlying cause [1]. Nutritional anemia remains a particularly challenging problem in underdeveloped countries, while in the developed world, rates have been substantially declining over the last decades [2]. In the United States, up to 5% of toddlers are anemic [3]. Prevalence rates in European toddlers vary between 3% and 48% and are significantly higher in Eastern Europe compared to Western countries [4]. 

Most commonly, iron deficiency anemia (IDA) is asymptomatic or causes minor signs and symptoms, while severe anemia presenting with pallor, irritability, lethargy, tachypnoea, and tachycardia is far less frequent [5]. If unrecognized, IDA can be associated with multiple deficits, including impaired neurodevelopment, growth, and immunity [6]. Screening for risk factors is recommended at regular pediatric check-ups. In case IDA is suspected, a complete blood count (CBC) must be obtained, revealing decreased hemoglobin (Hb) concentration for age with microcytosis and hypochromia [7]. Treatment is focused on appropriate doses and scheduling of oral iron and dietary modifications [4,7]. 

The aim of our study was to analyze epidemiological data and clinical profiles of children and adolescents referred to pediatric hematologists at a tertiary institution and evaluate underlying etiologies, laboratory workup, and treatment outcomes of these patients. Additionally, we investigated the reasons and rationale for further referral of anemic children from primary care settings.

## Materials and methods

We conducted an observational, cross-sectional study of patients referred to a pediatric hematologist at the Children's Hospital Zagreb, Croatia, from January 1, 2017, to December 31, 2021, with a diagnosis of IDA (International Classification of Diseases, Tenth Revision (ICD-10) D50.0-D50.9). Anemia was defined using the WHO 2011 criteria, as Hb levels below 110 g/L for infants from the sixth to 59th month, below 115 g/L for children from five to 11 years, and below 120 g/L for adolescent girls and 130 g/L for adolescent males [8]. Ferritin cut-off values of 12 µg/L for infants and children up to 59 months and 15 µg/L for children from five to 18 years were used to define iron deficiency [9]. The study was approved by the Ethics Committee, Children's Hospital Zagreb (approval number: 01-23/37-3-24) and conducted in accordance with the Declaration of Helsinki.

Electronic medical records were retrospectively analyzed, and epidemiological, clinical, and laboratory data were extracted and filled into designated tables. Children with incomplete data or incorrectly referred to as IDA were excluded. Epidemiological data included age, sex, and year of first referral. Information on the referring physician (primary care pediatrician, family medicine doctor, and other specialists such as gynecologists, pediatric gastroenterologists, and pediatric neurologists), diagnostic procedures carried out prior to tertiary institution consultation (CBC, biochemistry parameters), and therapeutic actions (iron supplementation therapy initiated, dosage, and type of product) were recorded. Clinical signs and symptoms attributed to anemia were collected (pallor, low energy levels, poor food intake, pica) [10]. Specific laboratory data (Hb, mean corpuscular volume (MCV), serum ferritin (SF)) were noted on three occasions (aforementioned primary care setting, first and last hematologist's visit). Additional workup (e.g., exclusion of gastrointestinal disease, thalassemia) planned by a hematologist was also documented, and etiology (dietary factors, gastrointestinal disease, menstrual bleeding, other) of sideropenia and sideropenic anemia was recorded. Information on oral iron therapy (dosage, product type, duration, compliance) prescribed by the hematologist was written down, as was the need for transfusion, parenteral iron therapy, and hospital admission. The differences regarding the number of visits, and diagnostic and therapeutic approaches among hematologists were analyzed. 

Descriptive statistical analysis was performed. For testing the hypothesis of independence for two categorical variables, chi-square or Fisher's exact test were used. A statistical significance was set at p<0.05. Statistical analysis was performed using R version 4.1.1 (R Foundation for Statistical Computing, Vienna, Austria).

## Results

**Figure 1:**
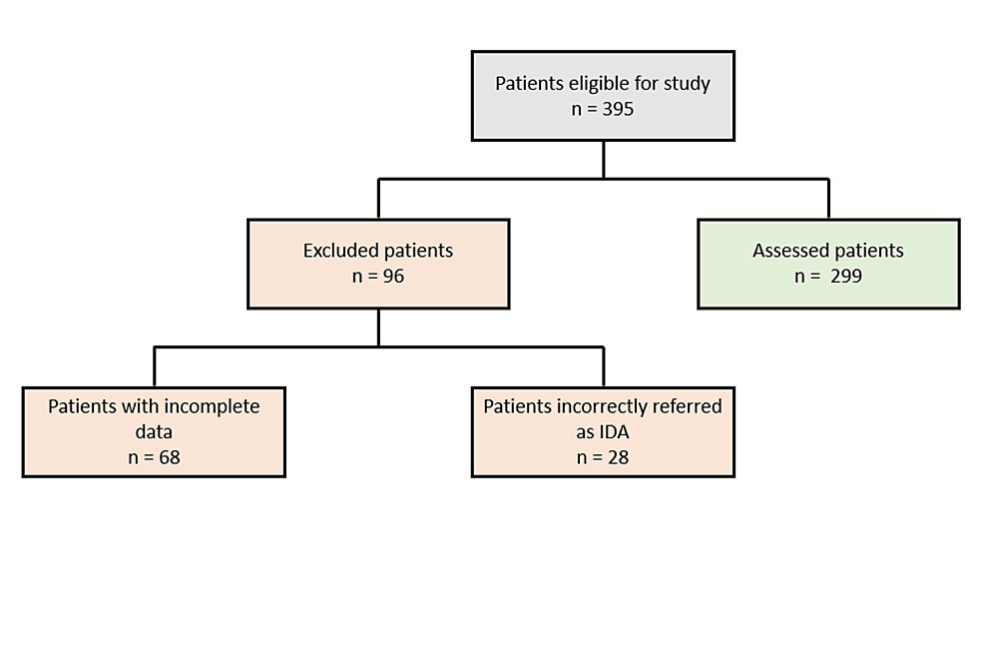
Patient flowchart. IDA: iron deficiency anemia

**Figure 2:**
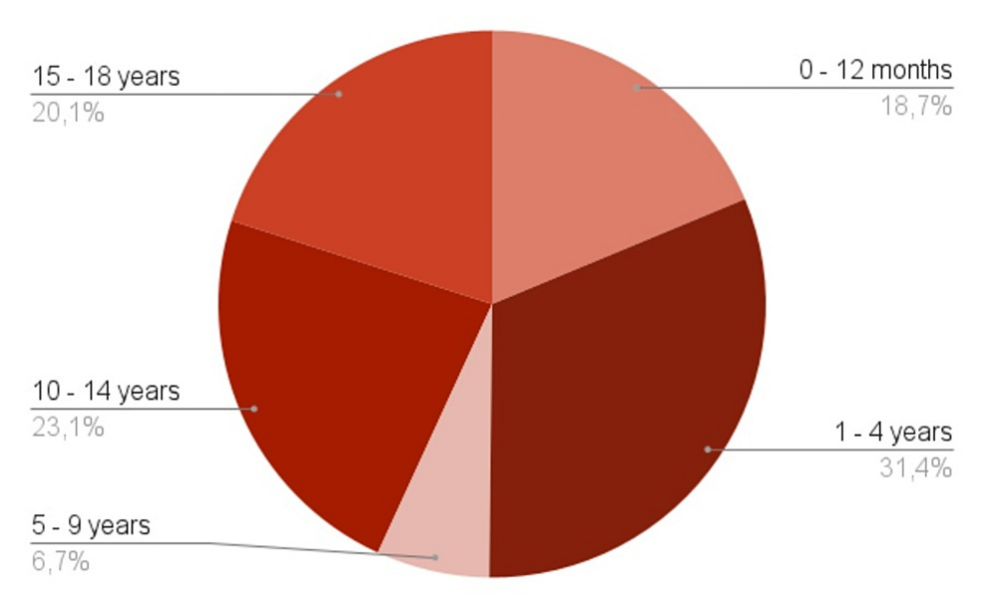
Patients' age group distribution. The data has been represented as %.

A total of 299 patients were included in the survey (Figure 1), of whom the majority were female (N= 156, 52.2%), predominantly of preschool and early adolescent age, with a median age of five years (Figure 2). 

**Figure 3:**
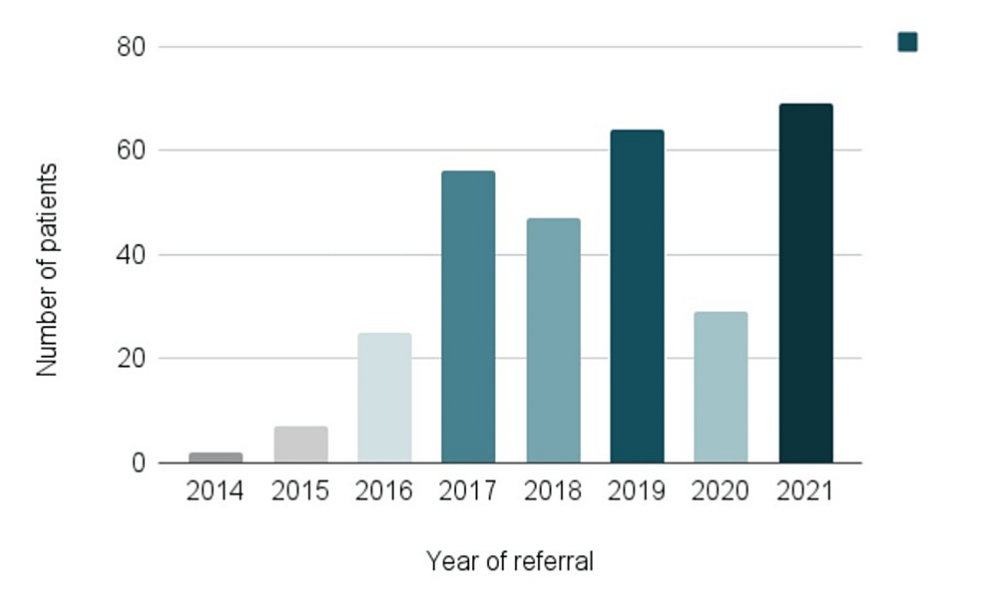
Year of referral. The data has been represented as N.

The greatest number of patients were referred to the hematologist’s office in the year 2021 (Figure 3).

Slightly more than 10% of patients were already in the hematologist’s care due to IDA in 2017, the initial year of our survey, and therefore included in the study. The patients were seen by seven hematologists, marked as No1-No7. The number of examined children per physician varied significantly, from five to 94, and more than half of the patients (N=177, 59%) were treated by hematologists No1 and No2. The children were referred mostly by the primary care pediatrician (N=129, 45.1%), followed by other specialists (N=88, 30.8%) and family medicine doctors (N=69, 24.1%). Results of basic laboratory tests performed at the primary and tertiary care centers are shown in Table 1. 

**Table 1:** Basic laboratory results, workup extent, and introduction of oral iron therapy in the primary and tertiary care settings.

Laboratory value		Primary care setting	Tertiary institution
Hemoglobin (g/L)	Mean	97.7	105
	Median	96	106
Mean corpuscular volume (fL)	Mean	71.4	72.9
	Median	71.8	74.5
Ferritin (µg/L), initial	Mean	-	15.1
	Median	-	7
Ferritin (µg/L), final	Mean	-	21.2
	Median	-	17.7
Diagnostics	Extended, N (%)	27 (11.2%)	184, (63.7%)
Oral iron initiated	Primary care pediatricians, N (%)	75 (58.1%)	-
	General practitioners, N (%)	30 (44.7%)	-
	Hematologists, N (%)	-	299, (100%)
	Other specialists, N (%)	-	56, (67.4%)

There was no statistically significant difference regarding the extent of diagnostics performed by different hematologists (p=0.925). Therapy was initiated in the primary healthcare setting in only half of the patients while all the children were treated with oral preparation by hematologists (Table 1). There was no significant difference in the frequency of prescribing iron therapy prior to the hematologist's consultation between primary care pediatricians and general practitioners (GPs), although other specialists were more likely to start iron therapy than family medicine doctors (p=0.019). The preferred prescribed preparation by the hematologists was Dextriferron (N=170, 67.7%) with a median dose Fe 5 mg/kg, during the mean period of 5.8 months (median 4, range 1-27 months). There was a notable difference in its prescription between different doctors, from 50% of cases by doctor No5 to 80% of cases by doctor No1. Far less commonly used iron preparations, each in about 6-8% of cases, were iron (II)-fumarate, iron (II)-sulphate, and ferrous glycine sulphate, while iron protein succinate was prescribed to only 3% of the patients. The precise dose of iron was specified in less than half of the cases (N=127, 42.5%). More than one-third of the patients (N=109, 36.5%) were non-compliant with iron medication according to the physicians' assessment.

In half of the patients, the hematologists did not specify the cause of IDA, while in the other half, the predominant etiology was menstruation (N=67, 22.5%). One-third of the patients (N=96, 32.4%) were symptomatic, significantly more often when menstrual bleeding was determined as the etiology of IDA (p<0.001). Screening for thalassemia was performed in approximately half of the patients (N=153, 51.2%), and seven children tested were diagnosed with concomitant beta-thalassemia (4.5%). The most common chronic diseases noted in our patients’ medical records were neurological disorders (N=17, 5.7%), followed by atopic dermatitis (N=8; 3%).

On average, the hematologist’s follow-up lasted for a year, with a median of four visits per patient. Every 10th child seen by the hematologist for IDA was admitted to the hospital (N=28), and 6.4% (N=19) required transfusion therapy. No statistically significant difference was found in the rate of recommended hospital admissions (p=0.628) or blood transfusions prescribed (p=0.729) by different hematologists. Parenteral iron was administered in only one patient. Due to the small number of hospital admissions, transfusions, and intravenous iron prescriptions, no statistical analysis among patients with different IDA etiology was possible. 

## Discussion

Given the relatively high global prevalence and the presumably negative impact of unrecognized IDA on children’s somatic, cognitive, and psychomotor outcomes, a comprehensive preventive and treatment approach is a major public health concern, even in developed countries. Strategies for IDA prevention, including primarily dietary measures, screening methods, and iron supplementation, differ between geographical areas, age groups, and risk factors contributing to ID. Recommendations include mostly infants and toddlers, in whom rapid growth demands high iron intake. While some organizations like the Spanish Association of Primary Care Paediatrics, the United Kingdom National Screening Committee, and the United States Preventive Services Task Force advise selective laboratory screening only, others, such as the American Academy of Pediatrics (AAP), encourage universal screening for IDA in all young children [11-15]. 

Data on IDA prevalence among healthy children in Croatia are lacking. According to a study of a tertiary institution in the Croatian town of Split, anemia was present in approximately 3% of hospitalized children, of which 87% was IDA, mainly present in children with respiratory tract infections [16]. Another cross-sectional study, conducted among a population of Croatian infants, found a rather high prevalence of IDA: 19.7%, 32.4%, and 16.4% at four months, six to nine months, and 12 months of age, respectively [17]. Croatian guidelines for prevention, screening, diagnosis, and treatment of IDA, proposed in 2019 by the Croatian Hematology Society and the Croatian Cooperative Group for Hematologic Diseases (CROHEM), are based on AAP recommendations. They suggest assessment of risk factors (prematurity, low birth weight, exclusive breastfeeding beyond four months of age, excessive cow’s milk consumption, low socioeconomic status) at regular check-ups at the age of four, 15, 18, 24, and 30 months, and three years, beyond which once yearly, focusing mainly on dietary habits. Universal laboratory screening is advised in late infancy, minimally by performing CBC, while the affirmative test of choice is SF. Further evaluation (peripheral blood smear, reticulocyte count, iron profile, and stool blood occult test) is required before initiating iron therapy in children >24 months of age or in severe IDA (Hb <70 g/L) [18].

Although the median age of anemic patients referred to our hospital was five years, indicating the need for extensive laboratory workup, most children had only Hb concentrations obtained before tertiary care visits. The non-compliance to the algorithmic approach could be explained by the poor availability of many biochemistry tests and cytological laboratories in primary healthcare, including SF concentration assay, which is, paradoxically, the cornerstone of IDA diagnostics. The fact that the median age of children referred to our institution was five years points out the possible neglect of this age group in which IDA risks are rarely investigated, unlike in infants. On the other hand, frequently present picky eating in preschoolers leads to sometimes profound nutritive deficiencies and symptomatic anemia. In addition, we believe that due to aversion, children of this particular age may often refuse to take prescribed iron, leading to poor compliance and lack of therapeutic effect, which troubles primary care physicians. Besides young children with nutritional anemia, the other most represented group was adolescent girls with menorrhagia, as per literature data [19]. 

The goal of IDA treatment is the replenishment of body iron stores. Numerous oral iron preparations vary in pharmaceutical forms and types of iron salts; divalent salts prevail over trivalent due to greater bioavailability. Oral iron preparations in Croatia are registered as medications or dietary supplements (iron bis-glycinate, lactate, fumarate, gluconate, saccharate, and pyrophosphate). Medications on the list of the Croatian Insurance Health Fund (CIHF) are currently iron (II)-fumarate and iron (III)-hydroxy polymaltose complex (Dextriferron), while the production of iron protein succinate recently ceased [18]. Ferrous sulphate, although globally the most prescribed iron supplement, with evidence of superior effectiveness over Dextriferron in children, is not on the CIHF list, but can be bought in pharmacies with a medical prescription [20]. Dextriferron, expectedly, being the cheapest and most accessible oral iron in Croatia, available as syrup feasible for use in small children, was the preferred prescribed formulation in our study. The median prescribed dose was in accordance with national guidelines (Fe 3-6 mg/kg body weight daily), and so was the treatment duration [18]. 

Gastrointestinal side effects of oral iron, such as abdominal pain, nausea, vomiting, constipation or diarrhea and metal taste in mouth, interfere with therapy adherence [21]. Other well-known causes for inadequate adherence are forgetfulness, caregivers’ inadequate concern and knowledge on IDA, allergies and problems assessing the public healthcare system [22]. Although reasons for poor compliance to iron medications were not investigated in our study, we believe, from our everyday experience, they are mainly gastrointestinal intolerance and caregivers’ unawareness of possible sequelae of IDA on child’s development, due to the lack of appropriate education. Previous studies observed better adherence to iron medications in children of older and more highly educated mothers [22]. 

Intravenous iron is rarely administered in children. Its use is usually limited to cases of severe anemia unresponsive to oral treatment such as that in malabsorption due to celiac disease, inflammatory bowel disease (IBD) or short bowel syndrome, and in iron-refractory iron deficiency anemia (IRIDA). Other potential causes may be substitution for transfusion in need of a quick recovery, end-stage renal disease, gastrointestinal intolerance, and non-adherence to oral medications. The main pitfalls of parenteral iron are common and sometimes life-threatening adverse reactions. Even though rare, possible anaphylaxis justifies hospitalization during intravenous iron administration in children [18,23,24]. New-generation preparations have a safer profile and are better tolerated and efficacious in children; however, the reluctance to use them persists [25]. The fact that only one patient was treated with intravenous iron confirms we still prefer transfusion therapy in cases of profound or refractory anemia. In addition, children receiving iron parenterally are mainly those with intestinal failure hospitalized at the gastroenterology ward and are therefore not included in these statistics. 

Being often overwhelmed by the number of patients referred for benign hematological conditions to our outpatient clinic, this study was principally initiated with the idea to investigate the rationale for further referral as well as diagnostic and therapeutic interventions carried out at the primary care level in children with IDA. The significant trend of increase in the number of hematologist consultations from 2015 onward, is the result of employing four new pediatricians in our department during the same year. Thus, the initial clinicians’ team doubled as well as the outpatient clinic’s capacities. On the contrary, the sudden drop of children referred to us for IDA in 2020 was the repercussion of coronavirus disease 2019 (COVID-19), which affected all aspects of pediatric care. During the early pandemic, as the result of strict lockdowns, our department’s work was oriented on emergencies and severely sick children in need of intensive treatment, while, although for a short period, many in-person visits, including those for mild anemia, were postponed if medically justifiable. We also believe that during 2020, preventive visits and IDA screening in primary pediatric care were negatively influenced by the COVID-19 pandemic. The reduction in the number of referrals was followed by the expected rebound in 2021. Although some hematologists were notably more involved than others in treating our IDA patients, there was almost a uniform diagnostic and therapeutic approach, including oral preparation selection, hospitalization, and transfusion indication. Most of the patients in our study had mild nutritional anemia, supported by the small number of hospital admissions and the need for transfusion therapy. Therefore, the majority of subjects could have been treated in the primary care settings with oral iron, which was initiated in only half of the children previous to tertiary care visits. 

The study's potential major limitation is the retrospective single-center design. In addition, there is potential referral bias, as half of the patients were already on iron therapy, and therefore the study does not reflect actual data on pediatric IDA in Croatia, for which further studies must be performed on the primary care level. 

## Conclusions

Despite the existence of national guidelines on childhood IDA, their implementation in primary healthcare often depends on doctors’ interest, knowledge of the topic, and the availability of diagnostic tests. Croatia’s primary healthcare network is deficient in pediatricians and GPs, who, overpowered by the amount of work, tend to seek subspecialists’ help more frequently. Nevertheless, a few pediatric oncologist-hematologists simultaneously treat children with malignancies and benign hematological diseases and are overwhelmed, as most cases of anemia are simple nutritive deficiencies that do not need further diagnostics and can be resolved by iron supplementation at the primary care level.

The fact that only about half of children with anemia had iron therapy initiated before a hematologist visit, unfortunately, indicates the unjustified fear of oral iron supplements and/or misdiagnosis, which we hope will decrease by pointing out evidence as obtained by our survey. In addition, current national pediatric residency programs include rotations on pediatric hematology wards with education on screening and prevention of IDA, which will hopefully add to better education of pediatricians on the topic. We strongly advise adherence to the guidelines’ algorithm whenever possible, with hematologist consultation only in cases of profound, acutely onset or persistent, refractory anemia, suspicious for an underlying condition.
